# Structural Effects of Metal Single-Atom Catalysts for Enhanced Photocatalytic Degradation of Gemfibrozil

**DOI:** 10.1021/acsanm.2c02859

**Published:** 2022-10-14

**Authors:** Vincenzo Ruta, Alessandra Sivo, Lorenzo Bonetti, Mark A. Bajada, Gianvito Vilé

**Affiliations:** Department of Chemistry, Materials, and Chemical Engineering “Giulio Natta”, Politecnico di Milano, Piazza Leonardo da Vinci 32, MilanoIT-20133, Italy

**Keywords:** photocatalysis, carbon nitride, heterogeneous catalysis, pollutant degradation, single-atom catalysis

## Abstract

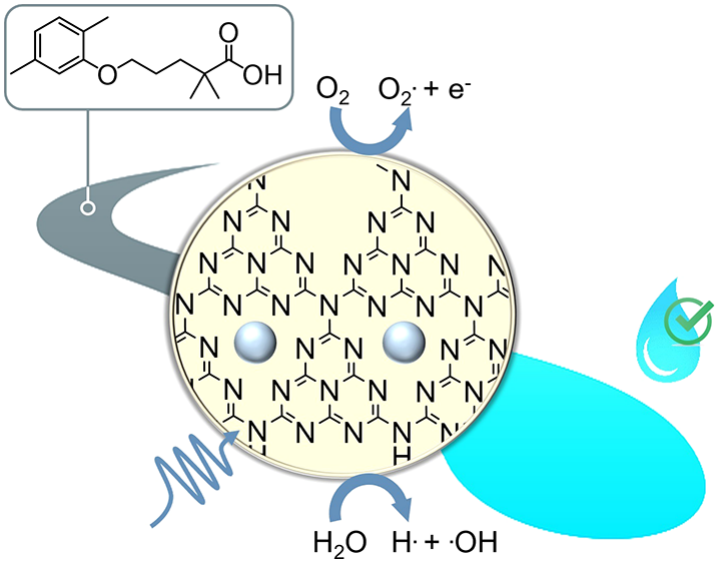

The development of efficient catalysts is a highly necessary but challenging task within the field of environmental water remediation. Single-atom catalysts are promising nanomaterials within this respect, but in-depth studies encompassing this class of catalysts remain elusive. In this work, we systematically study the degradation of gemfibrozil, a persistent pollutant, on a series of carbon nitride photocatalysts, investigating both the effect of (i) catalyst textural properties and (ii) metal single atoms on the contaminant degradation. Tests in the absence of the catalyst result in negligible degradation rates, confirming the stability of the contaminant when dispersed in water. Then, photocatalytic tests at optimal pH, solvent, and wavelength reveal a correlation between the support surface area and the degradation. This points to the role of carbon nitride surface nanostructure on gemfibrozil degradation. In particular, the use of silver on mesoporous carbon nitride single-atom catalyst (Ag@mpgC_3_N_4_) leads to an unprecedented degradation of gemfibrozil (>90% within 60 min). The possible degradation intermediates and products were identified by mass spectrometry and were inert by cytotoxicity evaluation. We anticipate that, with further refinement and customization, the carbon nitride catalysts reported herein may find broad applications for light-driven degradation of other contaminants of emerging concern.

## 1 Introduction

Water pollution caused by pharmaceuticals, personal care products, and metabolites is a major environmental problem because of the incremental consumption of drugs, which end up in aqueous effluents and are responsible for hazardous consequences to animals and ecosystems.^1,2^ To solve this challenge, heterogeneous photocatalytic processes are preferred^3−7^ on account of the facile catalyst separation and recyclability, which are key assets for large-scale industrial implementation of the technology.^9,10^ The lipid metabolism regulator gemfibrozil is one of those persistent and hazardous contaminants in Europe, Asia, and America.^8^ For its removal, the use of TiO_2_-based catalysts has been reported in the literature.^11^ However, this material leads to the incomplete degradation of the contaminant and to the formation of stable benzene derivatives that are potentially more harmful than the drug itself. Among alternative (non-TiO_2_ based) photocatalysts,^12,13^ carbon nitride (C_3_N_4_) polymeric structures have been found as a possible solution to the degradation of this and other similar compounds, including *p*-chlorophenol, bisphenol A, and imidacloprid.^14−16^ The excellent thermal and chemical stability and its semi-conductivity, with an optimal band gap of 2.7 eV (capable of absorbing in the range of visible light), make this material an attractive catalyst.^17−23^ These properties could be further enhanced by tuning the surface area of C_3_N_4_ through different synthetic methods, and through doping the material with metal and non-metal atoms, in order to create single-atom photocatalysts.^24−27^

Our previous investigation demonstrated the effectiveness of Ni-based single-atom catalysts based on C_3_N_4_ for the photodegradation of gemfibrozil.^28^ These findings inspired us to study the role of the catalyst support in order to anchor single atoms. In fact, it has been reported that nitrogen-rich carbon materials such as C_3_N_4_ could observably ameliorate the electron donor trend of carbon and maximize the exposure of isolated metal sites due to their high electrical conductivity, large specific surface area, and unique hollow structure, which makes it possible to better disperse metal atoms.^28^ However, C_3_N_4_ can exist in various nanoforms (*i.e.*, graphitic, exfoliated nanosheets, and mesoporous). Therefore, it is of great significance to rationally understand the support effect and design an efficient single-atom catalyst on the most appropriate carrier, exploring its application in environmental remediation.

In this work, we report the nanoengineering of C_3_N_4_ single-atom catalysts for gemfibrozil photodegradation, exploiting the role of surface area, metal type, and metal loading. Reaction conditions in the absence of any catalyst were evaluated first to probe the impact of different environments on the photodegradation rate. This was followed by a series of photocatalytic tests, where several types of C_3_N_4_ carriers (namely graphitic, nanosheet, and mesoporous C_3_N_4_) were applied in the absence and presence of metals. These tests allowed identification of optimal catalyst structures for gemfibrozil photodegradation.

## 2 Experimental Details

### 2.1 Preparation of C_3_N_4_ Carriers

**Figure 1:**
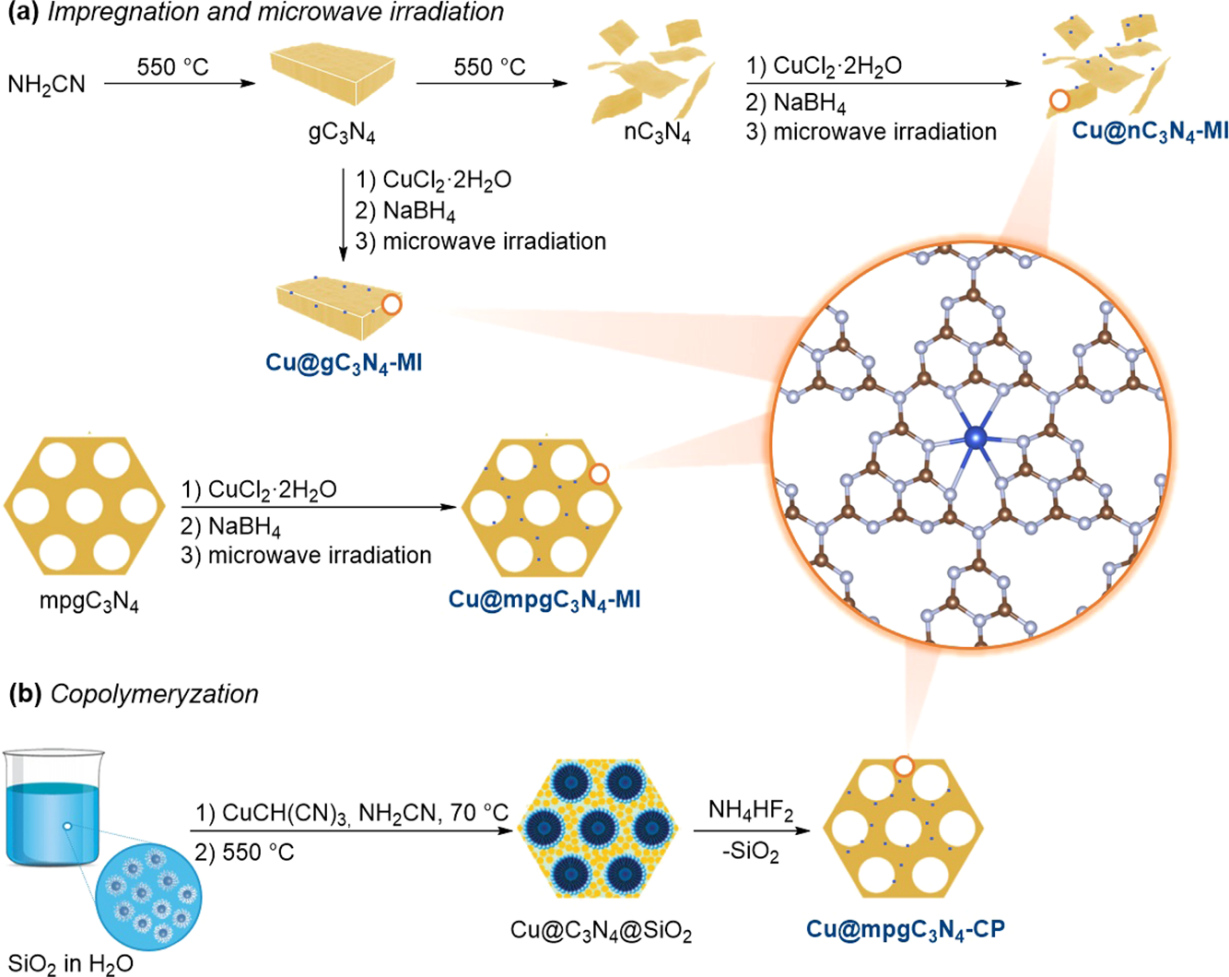
Illustration depicting the catalyst synthesis *via* microwave-assisted impregnation (a) and copolymerization (b), for the case of copper-based materials. The routes herein also apply to other metals.

Graphitic C_3_N_4_ (gC_3_N_4_), nanosheets of C_3_N_4_ (nC_3_N_4_), and mesoporous graphitic C_3_N_4_ (mpgC_3_N_4_) were prepared based on previously established procedures.^29−31^ The synthetic method is schematically depicted in Figure 1a. In brief, to obtain gC_3_N_4_, cyanamide (10 g, 0.23 mol; Sigma-Aldrich, 99%) was heated at 550 °C for 3 h, using a heating ramp of 10 °C min^–1^. To obtain nC_3_N_4_, gC_3_N_4_ was further treated at 550 °C for 3 h, using a heating ramp of 2 °C min^–1^. To prepare mpgC_3_N_4_, cyanamide (3.0 g, 0.07 mol) and a 40% aqueous dispersion of 12 nm SiO_2_ particles (7.5 g, Ludox HS40; Sigma-Aldrich) were mixed in a round-bottom flask and stirred at 70 °C for 16 h. The resulting mixture was heated to 550 °C (heating ramp: 2.2 °C min^–1^), and maintained at this temperature for 4 h. The brown-yellow powder was washed with a NH_4_HF_2_ solution (12 g in 50 mL of water; Sigma-Aldrich, 95%) and kept under stirring at room temperature for 24 h. The suspension was filtered, and the solids were washed three times with water and ethanol. Finally, the product was dried under vacuum at 60 °C overnight.

### 2.2 Single-Atom Catalysts *via* Microwave-Assisted Impregnation

To obtain the Cu-based single-atom catalysts, a solution of CuCl_2_·2H_2_O (0.011 g, 0.0007 mol; Sigma-Aldrich, 99%) in water (20 mL) was added to a fixed amount of gC_3_N_4_, nC_3_N_4_, and mpgC_3_N_4_ (0.5 g). The suspension was sonicated for 30 min and kept at ambient temperature for 12 h. The obtained material was treated with NaBH_4_ (1.5 g, 0.04 mol; Sigma-Aldrich, 99%) at 80 °C for 12 h, followed by 10 runs of microwave irradiation (2 min, 1000 W). The solids were filtered, washed three times with water and ethanol, and dried under vacuum at 60 °C overnight. The obtained catalysts were named Cu@gC_3_N_4_-MI, Cu@nC_3_N_4_-MI, and Cu@mpgC_3_N_4_-MI, respectively, where the suffix MI refers to “microwave-assisted impregnation”. The synthesis is also schematically represented in Figure 1a.

### 2.3 Single-Atom Catalyst *via* Copolymerization

Two single-atom catalysts were prepared *via* copolymerization, as shown in Figure 1b. A solution of CuCl_2_·2H_2_O (1.70 g, 0.01 mol) in water (10 mL) or AgNO_3_ (1.70 g, 0.01 mol) in water (10 mL) was added to a stirred solution of sodium tricyanomethanide (1.13 g, 0.01 mol; Sigma-Aldrich, 99%) in water (10 mL). The mixture was stirred for 3 h and filtered to separate the solids. The obtained solids were washed with water (3 × 10 mL) and dried in vacuum (7 mbar, 50 °C). Metal tricyanomethanide salts (105 mg, 0.00053 mol for copper(II) tricyanomethanide and 37 mg, 0.00015 mol of silver(I) tricyanomethanide) were mixed with cyanamide (3.0 g, 0.07 mol) and a 40% aqueous dispersion of 12 nm SiO_2_ particles (7.5 g, Ludox HS40; Sigma-Aldrich) and stirred at 70 °C for 16 h. The resulting mixture was heated to 550 °C (heating ramp: 2.2 °C min^–1^), and maintained at this temperature for 4 h. The brown-yellow powder was washed with a NH_4_HF_2_ solution (12 g in 50 mL of water; Sigma-Aldrich, 95%) to remove the silica template. The resulting suspension was filtered, and the solids were washed three times with water and ethanol. Finally, the product was dried under vacuum at 60 °C overnight. The obtained catalysts were named Cu@mpgC_3_N_4_-CP and Ag@mpgC_3_N_4_-CP, respectively, where the suffix CP refers to “copolymerization”.

### 2.4 Catalyst Characterization

Powder X-ray diffraction (XRD) was performed on a Philips model PW3040/60 X-ray diffractometer using Cu Kα radiation (λ = 0.15418 nm). Nitrogen physisorption measurements were performed after degassing the samples at 150 °C for 20 h using a Micromeritics 3Flex porosimeter at 77 K. The specific surface areas were calculated by applying the Brunauer–Emmett–Teller (BET) model to adsorption isotherms for 0.05 < *p*/*p*_0_ < 0.3 using the QuadraWin 5.05 software package. The pore size distribution was obtained by applying the quenched solid density functional theory model for N_2_ adsorbed on carbon with a cylindrical pore shape at 77 K. Elemental analysis (CHNS) was accomplished by combustion analysis using a Vario Micro device. An inductively coupled plasma optical emission spectroscopy (ICP-OES) study was performed using a HORIBA Ultra 2 instrument equipped with photomultiplier tube detection. X-ray photoelectron spectroscopy (XPS) was conducted using a Physical Electronics Instruments Quantum 2000 spectrometer with monochromatic Al Kα radiation generated from an electron beam operated at 15 kV and 32.3 W. All spectra were referenced to the C 1s peak of adventitious carbon at 284.8 eV. Scanning electron microscopy (SEM) micrographs were obtained using a Hitachi Tabletop SEM TM3030. Transmission electron microscopy (TEM) studies were performed using a double Cs-corrected JEOL JEM-ARM200F (S)TEM instrument operated at 80 kV equipped with a cold field emission gun. X-ray absorption spectroscopy (XAS) data were collected using synchrotron radiation. The spectra were recorded at the SuperXAS beamline of the Swiss Light Source at the Paul Scherrer Institute in Villigen, Switzerland. The obtained data were collected in the quick mode within 5 min using a Si(111) monochromator. The beam size at the sample position was 5 mm (horizontal) × 0.8 mm (vertical). For Ag and Cu L_3_-edge extended X-ray absorption fine structure (EXAFS) analysis, the oscillations were extracted using a spline smoothing method. The Fourier transform of the *k*^3^-weighted EXAFS oscillations and *k*^3^χ(*k*) from *k*-space to *r*-space was conducted in the range of 3–13 Å^–1^ for curve fitting analysis. The EXAFS data were analyzed using the Demeter software package. The ultraviolet photoelectron spectroscopy spectra were acquired with a He I (21.2 eV) radiation source. The detector was a combined lens with an analyzer module thermoVG (TLAM).

### 2.5 Catalytic Experiments

**Figure 2:**
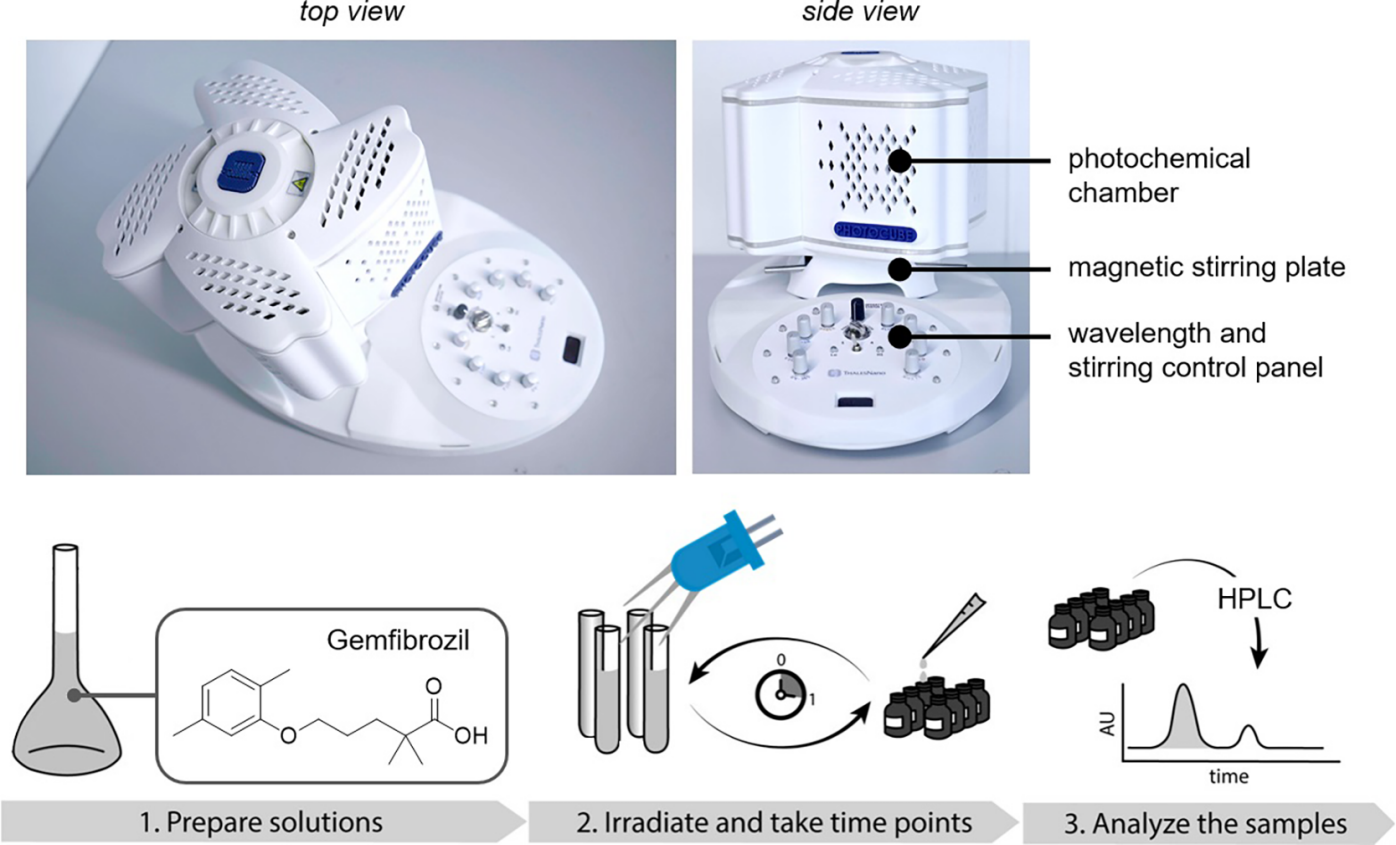
Photographs of the PhotoCube photoreactor (top, courtesy of ThalesNano). This compact system is the first of its kind offering seven wavelengths that can be used (even simultaneously) for a diverse set of batch and flow photochemical reactions. General sequence of the steps performed in each photocatalytic experiment (bottom).

Gemfibrozil (2.5 mg, 0.01 mmol, Sigma-Aldrich) was dissolved in 250 mL of solvent (water, acetonitrile, or xylene). When needed, the pH of the solution was adjusted using NaOH (1 M in water, Sigma-Aldrich) or HCl (37%, Sigma-Aldrich). The reaction was carried out at room temperature and with a reaction time of 60 min, using the PhotoCube reactor (ThalesNano, Inc). This compact system (Figure 2) is the first multi-wavelength instrument available for advanced photochemical applications, which enables to select and apply up to seven wavelengths, in addition to white, even simultaneously, and can be used to a diverse set of batch and flow reactions. At the end of each reaction, the catalyst was filtered, and the solvent was evaporated. For pH-adjusted reactions, the crude was diluted with brine (30 mL) and extracted with diethyl ether (30 × 3 mL). The organic phases were collected, dried over Na_2_SO_4_, and further evaporated. The resulting samples were diluted with acetonitrile (0.3 mg mL^–1^) and analyzed *via* high-performance liquid chromatography, using an Agilent 1200 instrument, equipped with an ultraviolet–visible detector (G1315D) set at 210 nm. The samples (10 μL) were injected directly onto a 250 mm × 4.6 mm Hypersil GOLD 5 μm 175 Å column (Thermo-Fisher) kept at 40 °C. The mobile phase was composed of a volumetric 60:40 acetonitrile/H_2_O mixture and was pumped at a total flow rate of 0.7 mL min^–1^. When conducting the analyses, a 45 min equilibration time was required before each sample injection.

### 2.6 Cytotoxicity Evaluation

The gemfibrozil-containing and the photochemically treated solutions were freeze-dried, then made up to the initial volume with culture media (CM). The test solutions were filter-sterilized (sterile syringe filter, pore size = 0.22 μm) before use. *In vitro* indirect cytotoxicity tests were performed in accordance with the ISO 10993-5:2009 standard. Dulbecco’s modified Eagle’s medium, supplemented with 1 mM sodium pyruvate, 10 mM HEPES buffer, 100 U mL^–1^ penicillin, 0.1 mg mL^–1^ streptomycin, 2 mM glutamine, and 10% (v/v) fetal bovine serum was prepared for cell culture and for the obtainment of the test solutions. L929 murine fibroblasts (ECACC, n. 85011425) were seeded in a 96-well culture plate (10^4^ cells/well in 100 μL of CM) and incubated in standard culture conditions for 24 h. Afterward, the culture medium was replaced with 100 μL/well of test solutions (*n* = 5 wells/sample type) and the plate was incubated for further 24 h in standard culture conditions. Cells cultured in CM were used as the control (CTRL, *n* = 5). Cell viability was assessed using the resazurin assay (Sigma-Aldrich). Fluorescence (λ_ex_ = 540 nm; λ_em_ = 595 nm) was measured with a Synergy H1 spectrophotometer. For each well, cell viability was calculated using to the following equation:
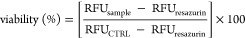
where RFU_sample_, RFU_resazurin_, and RFU_CTRL_ are the fluorescence of the sample (test solution), of resazurin, and of the control, respectively.

## 3 Results and Discussion

**Figure 3:**
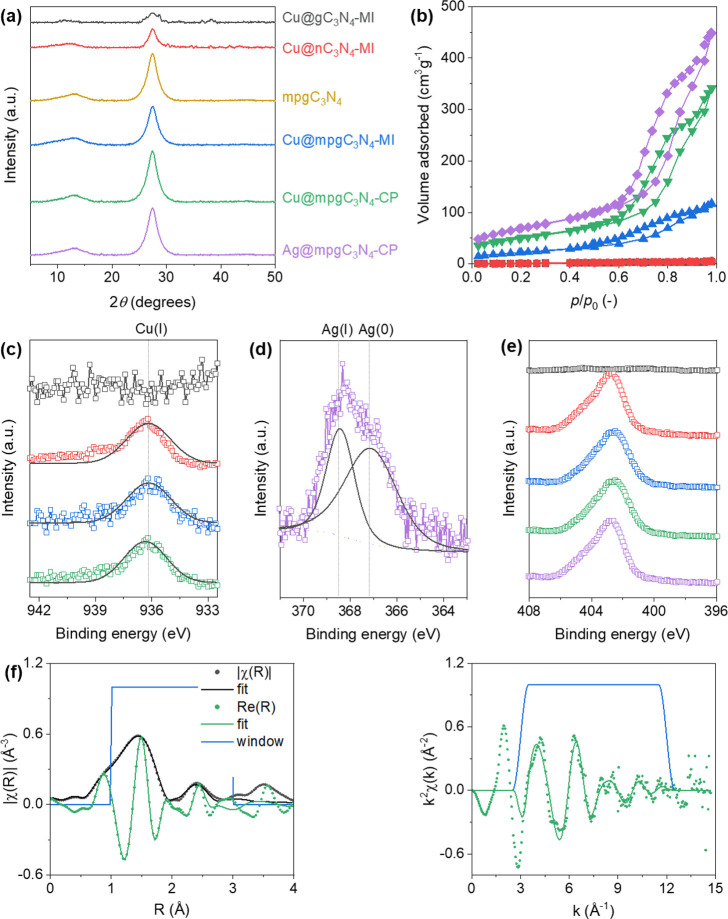
X-ray diffraction patterns (a). N_2_ physisorption isotherms (b). Cu 2p (c), Ag 3d (d), and N 1s X-ray photoelectron spectroscopy data of different catalysts (e). Extended X-ray fine structure spectroscopy data of Cu@mpgC_3_N_4_-CP (f). The color codes in (a) apply to (b)-(e) as well.

For the catalysts listed above, we evaluated the composition, purity, and crystallinity of the samples. Figure 3a shows the XRD patterns. Two typical diffraction peaks at 2θ = 13° and 28° are observed in all materials, corresponding to the (100) and (002) planes, respectively, which fit well with literature data.^32^ In particular, the weak peak at 13° corresponds to the *N*-linkage of the tri-*s*-triazine motif of the C_3_N_4_ carrier, whereas the peak at 28° shows the typical π–π stacking of aromatic structures in the support. No other peaks indexed to other phases and/or metallic clusters are observed.

N_2_ sorption experiments were used to analyze the porosity, pore distribution, and surface area properties of the catalysts. The adsorption isotherms (Figure 3b) highlight the nonporous morphology of gC_3_N_4_ and nC_3_N_4_, compared with the mesoporous network of mpgC_3_N_4_. This is confirmed by the surface area data in Table 1, which show minor values for g- and nC_3_N_4_ and larger areas for mesoporous materials, consistently with previously reported data.^33^ CHNS analysis and ICP-OES were done to calculate the elemental composition of the samples. The C/N ratio lies in the range of 0.61–0.69, which is close to the value of 0.75 for the theoretical C_3_N_4_ structure based on the tri-*s*-triazine pattern. The small discrepancies in the materials are usually assigned to polymerization defects, always observable in this kind of materials.^34^ The low values of H further verify the high level of polymerization and low residual protons within the structures. ICP-OES also confirms the successful incorporation of Cu and Ag.

**Table 1:** Elemental Composition, Surface Area, and Porosity Data of the Synthesized Materials

catalyst	C^a^(wt %)	N^a^(wt %)	H^a^(wt %)	C/N (−)	metal^b^(wt %)	*S*_BET_^c^(m^2^ g^–1^)
Synthesis Method: Impregnation and Microwave Irradiation						
gC_3_N_4_	34.84	56.65	1.10	0.61	-	5
nC_3_N_4_	32.15	47.92	2.12	0.67	-	7
mpgC_3_N_4_	31.90	48.75	2.43	0.65	-	157
Cu@gC_3_N_4_-MI	9.5	13.66	1.23	0.69	0.3	4
Cu@nC_3_N_4_-MI	11.47	17.76	1.71	0.65	0.3	7
Cu@mpgC_3_N_4_-MI	30.18	47.77	1.15	0.63	0.5	137
Synthesis Method: Copolymerization						
Cu@mpgC_3_N_4_-CP	31.37	48.78	2.2	0.64	0.5	241
Ag@mpgC_3_N_4_-CP	31.01	47.43	2.4	0.65	0.3	174

a CHNS data.

b ICP-OES data.

c BET method to the N_2_ isotherms collected at 77 K.

**Figure 4:**
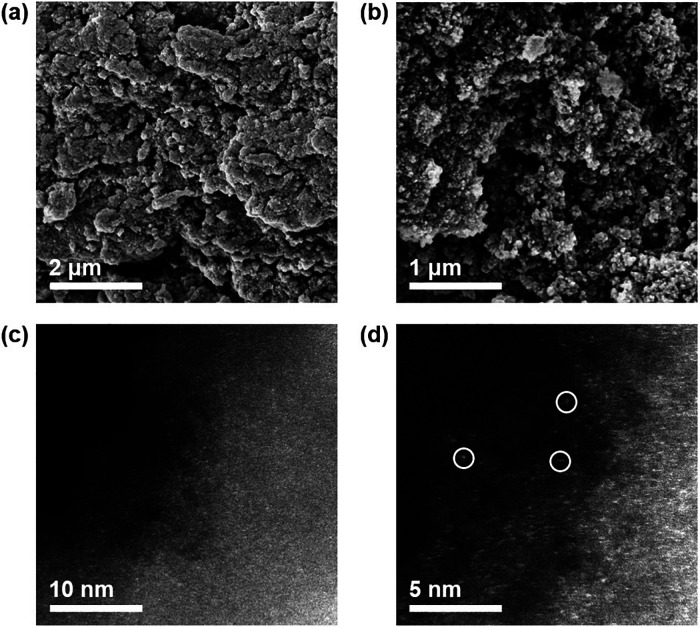
Micrographs of Cu@mpgC_3_N_4_-CP. SEM images at different magnification (a,b); high-resolution transmission electron microscopy of the same catalysts, highlighting the absence of metal clusters (c), and a higher magnification portion (d), evidencing the single-atom nature of the catalysts (circled).

Surface morphology and particle dimension was detected *via* SEM at different magnification, depicted in Figure 4a,b. The absence of nanoparticles, and the presence of isolated metal atoms was observed by high-resolution transmission electron microscopy (Figure 4c,d). To further support our observations of the single atoms, EXAFS spectroscopy of the Cu-containing materials was conducted (Figure 3f). From the fitting of the Fourier transform, the metal atom is surrounded by five neighboring ligands at an average distance of around 1.92 Å. Moreover, Cu resulted to be dispersed as single atoms, due to the absence of scattering contribution from Cu–Cu pairs.

XPS was carried out to elucidate the bonding and oxidation states of C, N, and metal atoms in representative catalysts (Figure 3c–e). The XPS C 1s spectra present two main prominent peaks, deconvoluted in several others, which point to the carbon hybridization states in the carrier. The peak at 284.8 eV is assigned to both adventitious carbons and C-sp^2^, typical for electron-rich C atoms. The peak at 285.9 is assigned for C-sp^3^ atoms and the peak at 288.4 eV is related to N–C=N carbons in the aromatic structure. In the N 1s spectra, the main peak can be deconvoluted into three principal peaks, the first at 398.4 eV, corresponding to the pyridinic nitrogen (C=N–C), the second at 399.4 eV, corresponding to tertiary nitrogen (C=N–C_3_), and the third at 401.4 eV, related to primary amine groups. In the Cu 2p spectra, there are two principal peaks corresponding to Cu 2p_3/2_ and Cu 2p_1/2_ for Cu^2+^ centered at 932 and 939 eV, , respectively, and sharp satellite peaks, confirming the abundant presence of the metal in this oxidation state. Moreover, the peak at 935 eV is assigned to metallic Cu, confirming the presence of the metal also in its elemental form. Ag 3d spectra present two significant peaks at 368.4 and 367.2 eV, which are characteristic of Ag^+^ and Ag^0^, respectively. Surface metal amounts by XPS were in line with the total metal content calculated *via* ICP-OES, evidencing a well distribution of the metal site on the surface.

Degradation experiments in the presence of visible light but with no catalyst were initially carried out to identify the best conditions for gemfibrozil photodegradation (Table 2). These tests were useful to explore the influence of pH, solvents, reaction mixture, and wavelength of the irradiation source. Investigating the effect of pH, tests in acidic, neutral, and alkaline media showed no degradation of the drug, which confirmed the relative stability of the molecule in both deprotonated and neutral forms. The presence of other elements, that is, inorganic salts, mimicking a more complex water system, showed no relevant influence, as no degradation occurred even in these conditions. The only degradation observed in this series of experiments was found using a different solvent, *p*-xylene, which resulted in a degradation of <5%. However, the slight enhancement of the degradation rate suggested the potential to achieve photodegradation of the compound *via* tuning the incident wavelength. Thus, UV light-driven tests were carried out, resulting in an enhanced but incomplete degradation of the compound, near 50%.

**Table 2:** Control Non-catalytic Experiments for the Photocatalytic Degradation of Gemfibrozil^a^

entry	pH	solvent	light	catalyst	degradation (%)
1	1	water (+HCl)	blue, visible	no	no
2	4.5	water (+HCl)	blue, visible	no	no
3	7	water	blue, visible	no	no
4	14	water (+NaOH)	blue, visible	no	no
5	7	xylene	blue, visible	no	3
6	7	water	UV	no	50
7	7	water	blue, visible	no	no

a Reaction conditions: *C*_0(Gemfibrozil)_ = 10 mg L^–1^, *t* = 60 min, room temperature, and wavelength λ = 457 nm for blue visible light, 365 nm for UV light.

**Figure 5:**
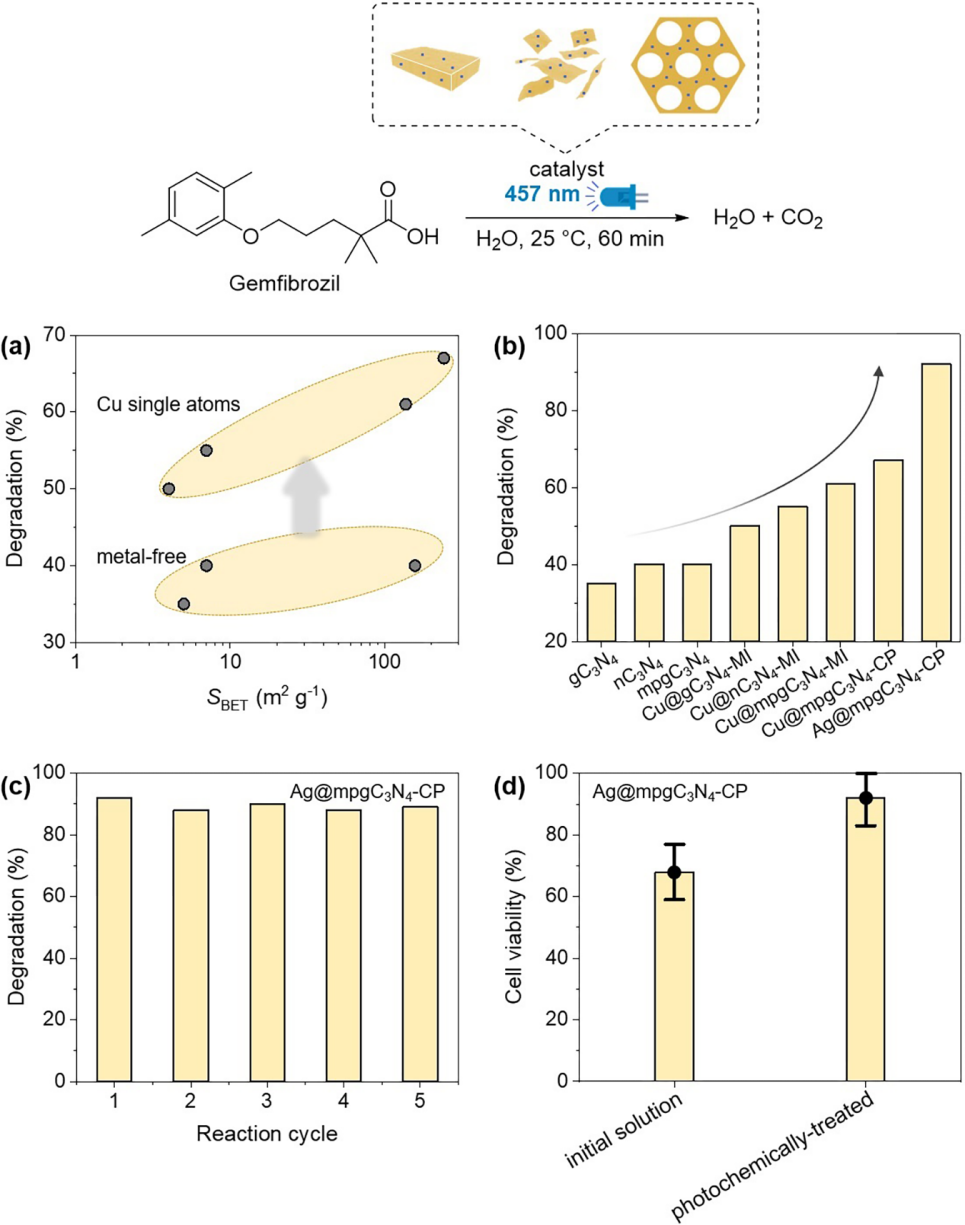
Photocatalytic degradation of gemfibrozil. Effect of the surface area for metal-free and Cu-based materials (a), evaluation of the degradation rate using several C_3_N_4_ catalysts (b), stability test over five reaction cycles (c), and cytotoxicity evaluation of the photochemically treated solution (d). Reaction conditions for all the catalytic tests: *C*_0(Gemfibrozil)_ = 10 mg L^–1^, *t* = 60 min, catalyst amount 0.006% mol (corresponding to 15 mg for metal-free samples), room temperature, λ = 457 nm, and solvent = water.

To push the degradation rate using visible light, we decided to use C_3_N_4_-based catalysts (Figure 5a). Metal-free C_3_N_4_ materials degraded gemfibrozil with moderate yields, with mpgC_3_N_4_ being more efficient than g- and nC_3_N_4_. These results confirmed the effectiveness of C_3_N_4_ to facilitate the energy transfer from light to the substrate. This key role is undoubtedly played by the C_3_N_4_ structure, capable of absorbing visible light and conveying electrons for the generation of radicals to partake in oxidative reactions. Given that the three supports have similar composition, the difference in degradation values produced by the three metal-free supports (gC_3_N_4_ < nC_3_N_4_ < mpgC_3_N_4_) can be related to the increase of surface area in the three structures. Differently from the two-dimensional gC_3_N_4_, in the nC_3_N_4_ the assembly of stacked graphitic planes results in a 3D material with a higher surface area. This increase is more accentuated in mpgC_3_N_4_, where the presence of mesopores, created by silica etching in the synthetic procedure, allows for higher values of the surface area to be attained.

We also decided to compare C_3_N_4_ materials with single-atom Cu and Ag functionalities. In general, the presence of the metal significantly speeds up the reactions, due to the creations of joint electronic structures that efficiently modify the band gap of the materials. This modification results in a considerable broadening of the light responsive range and in the enhancement of charge separation. Results of these experiments are shown in Figure 5b.

Also, for the cases including the metal single atoms, the importance of the surface area was confirmed by adopting several Cu-based materials with comparable metal loading (from 0.3 to 0.5 Cu wt %) but different surface areas (from 4 to 241 m^2^ g^–1^) (Figure 5b). We observed an exponential correlation between the degradation and the increased surface area. This outcome could be explained through consideration of the enhanced contact between light, catalyst, and the solution, in a material with an extended surface area and pore volume such as Cu@mpgC_3_N_4_-CP (*S*_BET_ = 241 m^2^ g^–1^ and *V*_pore_ = 0.69 cm^3^ g^–1^). Moreover, the adoption of different synthetic techniques (microwave irradiation and copolymerization) to produce two Cu@mpgC_3_N_4_ materials with comparable metal loading, also resulted in a major degradation for the catalyst with the material having the higher surface area, namely Cu@mpgC_3_N_4_-CP. Interestingly, the Ag-based sample resulted in an enhanced degradation due to the presence of a more porous surface. Additionally, the oxidative reactions in the presence of the Ag-based catalyst appear to be favored, due to the enhanced availability of this metal to accept electrons and thus to carry out oxidative reaction pathways.^35^ In this case, we could reach >90% degradation of gemfibrozil, within a 60 min reaction time frame. It should be highlighted that such degradation takes place in the presence of visible light and is among the highest degradation rates of gemfibrozil reported in the literature within this region (Table 3).

**Table 3:** Literature Precedents for the Visible Light Photocatalytic Degradation of Gemfibrozil^a^

catalyst	conditions	degradation (%)	reference
no catalyst	visible light (λ = 420),*T* = 30 °C	4	(8,9)
TiO_2_/C-dots	visible light (λ = 420),*T* = 30 °C	40	(11)
TiO_2_ P25	visible light (λ = 420),*T* = 30 °C	10	(11)
TiO_2_ anatase	visible light (λ = 420),*T* = 30 °C	8	(11)
TiO_2_/C-dots	visible light (λ = 420),*T* = 30 °C	37	(11)
saNi@nC_3_N_4_^a^	visible light (λ = 420),*T* = 30 °C	63	(28)
Ag@mpgC_3_N_4_-CP	visible light (λ = 457),*T* = 25 °C	92	this work

a Ni single-atom catalyst based on nanosheets of C_3_N_4_. The effect of the carrier was not evaluated in this work.

**Figure 6:**
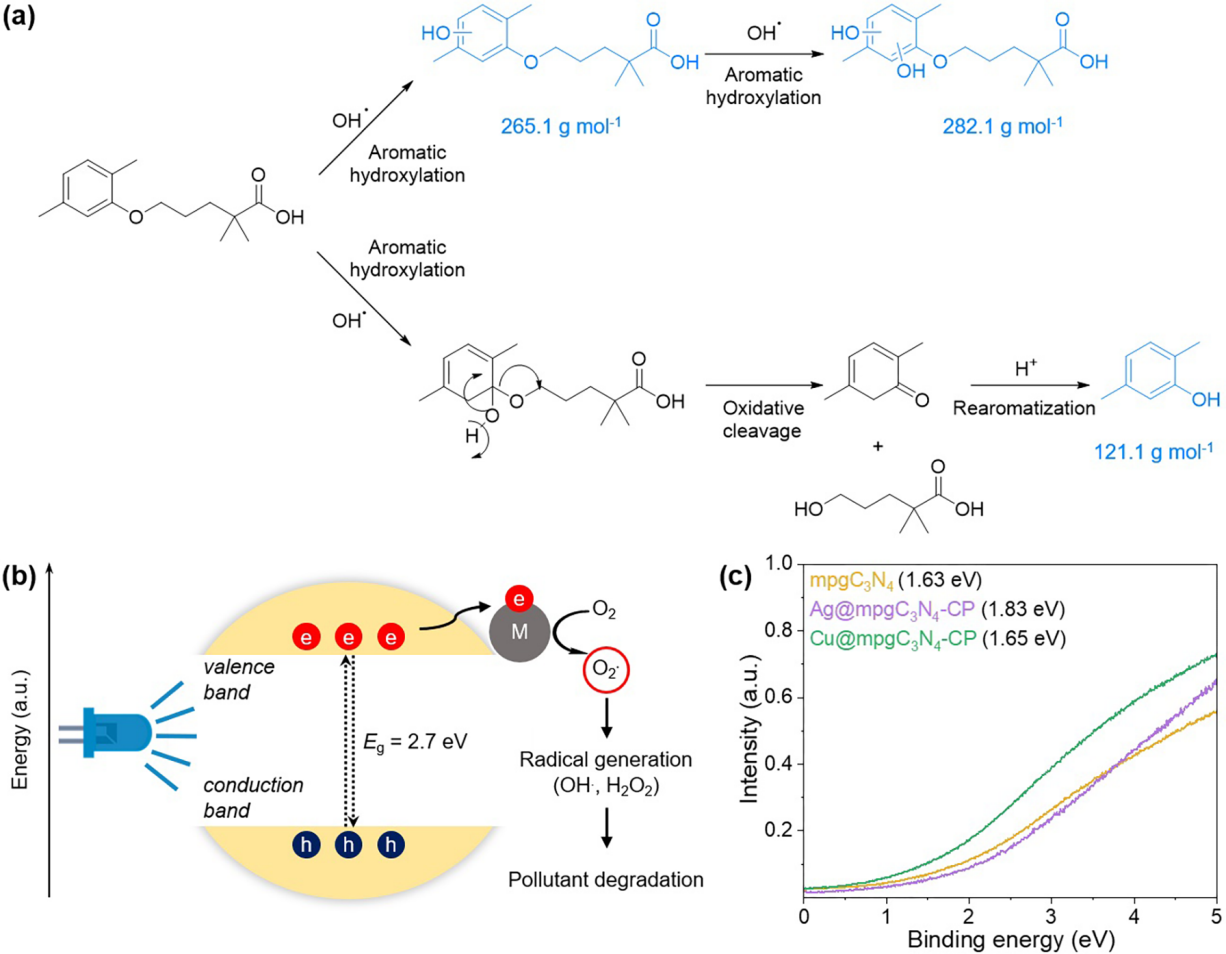
Proposed mechanism for the degradation of gemfibrozil over single-atom catalysts and, in blue, products obtained from the decomposition and detected *via* mass spectrometry (a), mechanism of visible light-mediated catalyst activation (b), and ultraviolet photoelectron spectroscopy of metal-free, copper, and silver-loaded catalysts, with the values of the valence band edge (c).

After evaluating the catalytic performance of the different catalysts, a photostability test on the best performing material (*i.e.*, Ag@mpgC_3_N_4_-CP) was carried out over five reaction cycles, demonstrating optimal stability and recyclability (Figure 4c). The hypothesis of the oxidative mechanism was confirmed by electrospray ionization-mass spectrometry analysis on the resulting mixture, through which we evaluated the degradation products formed. The major products resulting from the oxidative cleavage pathway on the aromatic moiety and the aromatic hydroxylation pathway can be noted in Figure 6a. These tests suggest an oxidative mechanism as the principal route for gemfibrozil degradation. In summary, the C_3_N_4_ photocatalytic mechanism leads to the generation of reactive oxygen species from the aqueous media, for example, OH^•^ or O_2_^•–^, *via* the transfer of excited electrons to the metal centers, which then partake in the degradation of gemfibrozil (Figure 6b,c).^36^ These species are well-known to facilitate oxidative chemistry. Moreover, oxy-radical generation is further enhanced by C_3_N_4_-catalyzed *in situ* formation of H_2_O_2_ that assists in the oxidation of aromatic moieties.^37^ To assess the toxicological profile of the reaction mixture, *in vitro* indirect cytotoxicity analyses were performed (Figure 5d). A low cell viability (68.5 ± 9.9%) can be observed for the non-treated mixture, which suggests cytotoxicity in accordance with the ISO 10993-5:2009 standard. In this regard, it has been reported how fibrates, such as gemfibrozil, can lead to oxidative stress in cultivated cells by enhancing β-oxidation of lipids.^38^ In addition, the toxicity of gemfibrozil could also be related to non-specific mechanisms of action (*e.g.*, non-specific disturbance of membrane integrity and functioning), lastly resulting into non-specific cytotoxicity and necrosis.^39^ Overall, the results obtained in this work are in line with literature data, which indicate significant dose- and time-dependent cytotoxicity of gemfibrozil, albeit on different cell types.^39,40^ Conversely, the photochemically treated solution displays no cytotoxic effect on L929 cells (92.0 ± 8.6% cell viability), suggesting nearly a complete detoxification. This is due to the low toxicity of the reaction products and to the complete degradation of the pharmaceutical pollutant that is in fact the more toxic component of the mixture.

## 4 Conclusions

In conclusion, the role of the reaction conditions, photocatalyst support, and metal single atom within C_3_N_4_ have been studied within the context of gemfibrozil photodegradation. This expands on our previous work, where Ni-based single-atom catalysts were found to be active in the degradation of gemfibrozil, using a nanosheet-based C_3_N_4_. Various C_3_N_4_ nanomaterials have been now produced, characterized, and tested, evaluating the effect of catalyst nanostructuring on the rate of photodegradation. Particularly, photocatalytic reactions carried out using different supports (g-, n-, and mpgC_3_N_4_) showed a correlation between the surface area and the contaminant degradation rate. The insertion of the metal in the C_3_N_4_ network resulted in an enhanced degradation with respect to the bare metal-free support, wherein the Ag-based mesoporous single-atom photocatalyst yielded the maximum rate of photodegradation, among the metal-doped materials studied. This work paves the way for the optimization of photocatalytic processes using metal-based single-atom catalysts and for future applications in environmental water remediation.
